# “The care is the best you can give at the time”: Health care professionals’ experiences in providing gender affirming care in South Africa

**DOI:** 10.1371/journal.pone.0181132

**Published:** 2017-07-12

**Authors:** Sarah Spencer, Talia Meer, Alex Müller

**Affiliations:** Gender Health and Justice Research Unit, University of Cape Town, Cape Town, Western Cape, South Africa; Pontificia Universidade Catolica do Rio Grande do Sul, BRAZIL

## Abstract

**Background:**

While the provision of gender affirming care for transgender people in South Africa is considered legal, ethical, and medically sound, and is—theoretically—available in both the South African private and public health sectors, access remains severely limited and unequal within the country. As there are no national policies or guidelines, little is known about how individual health care professionals providing gender affirming care make clinical decisions about eligibility and treatment options.

**Method:**

Based on an initial policy review and service mapping, this study employed semi-structured interviews with a snowball sample of twelve health care providers, representing most providers currently providing gender affirming care in South Africa. Data were analysed thematically using NVivo, and are reported following COREQ guidelines.

**Results:**

Our findings suggest that, whilst a small minority of health care providers offer gender affirming care, this is almost exclusively on their own initiative and is usually unsupported by wider structures and institutions. The ad hoc, discretionary nature of services means that access to care is dependent on whether a transgender person is fortunate enough to access a sympathetic and knowledgeable health care provider.

**Conclusion:**

Accordingly, national, state-sanctioned guidelines for gender affirming care are necessary to increase access, homogenise quality of care, and contribute to equitable provision of gender affirming care in the public and private health systems.

## Background

Globally, there are many crucial gaps in research and knowledge of the health of transgender people [1]. In South Africa, despite gender affirming care (GAC) being, in accordance with international standards and the South African National Health Act [2], “legal, ethical medical practice” [3], and such services being—theoretically—available in both the private and public sectors, access remains severely limited and unequal. As there are no national policies or guidelines, little is known about how individual health care professionals (HCPs) provide GAC. In an effort to fill this gap, this article presents original research with HCPs in South Africa to explore their knowledge, practices and constraints in the provision of GAC to transgender individuals in the absence of national policies or guidelines.

### Gender affirming care

The term ‘transgender’ has become an umbrella term for people who experience incongruence between the sex assigned to them at birth and their gender identity. Those who are gender diverse (that is, individuals who experience a difference between their gender identity, expression, or role and social norms conferred upon that individual’s sex), who experience gender incongruence, and either temporarily or permanently live in their identified gender with or without pursuing GAC can all identify as ‘transgender’ or ‘trans persons’ [4, 5]. The diversity of lived experiences of trans persons and the corresponding variety of medical needs has traditionally posed challenges for health authorities in setting diagnostic criteria and guidelines for providing GAC, although agreement on treatment standards has been achieved internationally [6]. While not all transgender individuals will pursue GAC, for those who do, it can be integral to realizing their gender identity, both personally and socially.

GAC works towards alleviating the potential distress or dysphoria of trans people through exploring and locating a gender identity that is suited to each individual [6]. GAC usually takes the form of medical interventions (which are discussed in the following section). Whilst relying on medical skills and technologies, and access to healthcare systems, the relationship between gender diversity (including trans identities) and biomedicine is a fraught one. Within medical discourses and health systems, gender diversity has been regarded as a mental health condition since the 1980s, including being listed in the International Classification of Diseases (ICD) [7] and the Diagnostic and Statistical Manual of Mental Disorders (DSM) [8, 9]. Trans activists have consistently asserted that gender outside of the strict male/female binary is simply a part of human gender diversity, is not an illness, and ought to be understood outside of biomedical frameworks [10]. To this end, over the last decade, trans activists have advocated for the removal of gender diversity-related diagnoses from diagnostic manuals, culminating in changes to the 11^th^ revision of the ICD. In the suggested forthcoming ICD-11, a gender diversity-related diagnosis will be maintained, however, it will be moved out of the mental illness chapter, and will be rephrased to ‘gender incongruence’ rather than ‘transsexualism’ [11]. It is hoped that this configuration will facilitate access to the medical interventions necessary for GAC within existing health systems, but also reduce pathologising language and stigma.

Currently, however, individuals who experience significant distress and wish to access GAC are frequently assigned the ICD-10 diagnosis of ‘transsexualism’, listed under the category of ‘gender identity disorders’ (GID)—“a desire to live and be accepted as a member of the opposite sex, usually accompanied by a sense of discomfort with, or inappropriateness of, one’s anatomic sex and a wish to have hormonal treatment and surgery to make one’s body as congruent as possible with the preferred sex” [7]—or the DSM-V diagnosis of ‘gender dysphoria’—“a marked incongruence between one’s experienced/expressed gender and assigned gender, of at least 6 month’s duration” where the “condition is associated with clinically significant distress or impairment in social, occupational, or other important areas of functioning” [8]. Both the World Health Organization (WHO) and the American Psychiatric Association’s (APA) diagnostic guidelines indicate that relief of these symptoms is positively associated with changes to the primary and/or secondary sex characteristics as a means of diminishing the level of incongruence with the individual’s gender identity [12].

### Internationally recognized standards of care

There are various psychological and physiological medical approaches that trans persons can pursue as GAC. The World Professional Association for Transgender Health (WPATH), in the seventh edition of its Standards of Care (SOC-7), outlines its recognized treatment options for those seeking care for gender dysphoria. These are inclusive of:

*changes in the gender expression and role (which may involve living part time or full time in another gender role*, *consistent with one’s gender identity); hormone therapy to feminize or masculinize the body; surgery to change primary and/or secondary sex characteristics (e*.*g*. *breasts/chest*, *external and/or internal genitalia*, *facial features*, *body contouring); psychotherapy (individual*, *couple*, *family*, *or group) for purposes such as exploring gender identity*, *role and expression; addressing the negative impact of gender dysphoria and stigma on mental health; alleviating internalized transphobia enhancing social and peer support; improving body image; or promoting resilience* [6].

Due to the highly individualized nature of each person’s lived experience, trans persons might variously choose to pursue all, some, or none of these paths of care.

### Medical necessity of gender affirming care

Despite the individualized nature of GAC, however, it is a clinically recognized necessity for those who wish to align their body to their identified gender. This necessity has been acknowledged by the WHO, APA, WPATH, and various health districts around the world. For example, the Royal College of Psychiatrists uses the SOC-7 to inform its United Kingdom standards of care [13], and the American Medical Association (AMA) shares WPATH’s position that GAC is neither cosmetic nor experimental and advocates for coverage of associated care by insurers, since “GID, if left untreated, can result in clinically significant distress, dysfunction, debilitating depression, and for some people without access to appropriate medical care and treatment, suicidality and death” [14]. Likewise, the APA’s official position on access to GAC is that it “opposes categorical exclusions of coverage for such medically necessary treatment when prescribed by a physician” [15].

While a diagnosis of GID/GD would suggest that poor health amongst transgender populations is a result of internal distress and discomfort with their gender incongruence, studies show that it is the social environment that individuals navigate in their daily lives which most significantly affects their health and wellbeing [16]. Accordingly, GAC–surgery in particular–is recognised as improving social recognition and reducing experiences with distress and dysphoria [12].

### Gender affirming care in South Africa

The South African post-apartheid state enacted a multitude of laws and policies to prevent discrimination and ensure the health of its people. The Constitution guarantees every citizen: the enjoyment of rights regardless of one’s “race, gender, sex, […] sexual orientation, age, […]”; the respect and protection of their inherent dignity; the right to life; freedom and security of their person including protection from inhumane and degrading treatment and the preservation of “bodily and psychological integrity,” including sovereignty in decisions regarding reproduction and control over the body; and the right to access healthcare services [17]. Case law has interpreted this as including protection based on gender identity [18, 19].

Access to GAC, however, is hindered by the realities of the South African health system. South Africa has both a public health system–of about 400 hospitals and 4000 primary care facilities [20], and, in line with state policies, a private system consisting of general practitioners (GPs) and about 200 hospitals, which are largely funded by private medical schemes [21].

There is significant inequity between the two sectors: just thirty per cent of the country’s doctors work in the public health system responsible for providing care to the overwhelming majority of the population who do not have private health insurance (over 40 million individuals) and, despite the already high concentration of doctors in the private sector, public sector doctors have lately acquired the ability to also work a portion of their time privately [22].

In addition to the structural inequality, the attitudes of HCPs toward transgender and gender diverse people present a considerable barrier to access to care, including GAC. Whilst there is little documentation of transphobia in the healthcare system specifically, trans advocacy organisations provide important first person accounts of gender-based discrimination [23, 24], and a recent study on heteronormativity in healthcare facilities demonstrates that gender diverse people experience severe and specifically gender-bias motivated discrimination by HCPs in South Africa [25].

The aim of this study was to determine how HCPs in South Africa provide GAC in the absence of national guidelines and, from this acquired understanding, to evaluate the potential role for national guidelines to assist in their work. Specifically, the study examines what GAC services are available in South Africa and where; which tools HCPs employ to inform their provision of GAC and how this impacts upon clients; clients’ ability to access services as well as HCPs access to other GAC-providing professionals; and the diverse challenges of providing GAC in South Africa.

## Materials and methods

### Study design

Based on an initial policy review and service mapping, the study employed semi-structured interviews with a snowball sample of twelve HCPs between December 2014 and February 2016. The authors are based in a South African research unit that works specifically on policy development and evidence-based advocacy for gender, sexual health and reproductive rights, and violence prevention in the South African and Southern African context. AM in particular has worked in the field of transgender health in South Africa for several years, and directed the team toward the relevant health policies. In addition, we examined all healthcare policy, including National Strategic Plans and treatment guidelines, on the website of the South African National Department of Health, and consulted other health policy experts as well as organisations working in transgender health and rights advocacy. South Africa experienced a radical policy change from 1991 due to the repeal of many apartheid laws and, subsequently, the implementation of laws that would govern the country under the new democratic dispensation. We reviewed all Department of Health policies since 1991 for content on GAC. For the service mapping, AM provided the initial database of HCPs, which we cross-checked against a list compiled by Gender Dynamix, the largest transgender advocacy organisation in South Africa. Additionally, we asked all participating HCPs if they were aware of GAC services provided elsewhere in the country, and searched newspaper articles about transgender health-related topics published within the last 10 years for information about HCPs that we may have missed.

### Sampling and recruitment

Through the policy review for this study, prior work with transgender support organizations and other HCPs, and media reports, we compiled a database of clinicians providing various aspects of gender affirming care. We identified eight professionals across the country, which covered the spectrum of GAC including a surgeon, clinical social worker, clinical psychologist, psychiatrist, sexologist, endocrinologist and GP, and worked in either the private or public health system, or both. Another five HCPs were referred to us by participating HCPs (Table 1). Because GAC is seen as highly specialized and is provided by a small group of professionals as identified through our database, this sample is not only indicative of the scope of expertise available but also captures the vast majority of GAC-providing HCPs in the country—although there may be GPs providing ad hoc services. Our recruitment goal was to interview all HCPs currently providing GAC in South Africa. To our knowledge, we succeeded in interviewing most HCPs working in the tertiary public sector, and in private.

**Table 1:** Interviewees.

Interview Code	Area of Specialisation	Sector	Province
HC001	Paediatric Endocrinology	Public	Western Cape
HC002	Child and Adolescent Psychiatry	Public & Private	Western Cape
HC003	Clinical Social Work	Public & Private	Western Cape
HC004	Surgery	Public & Private	Western Cape
HC005	Clinical Sexologist	Private	Gauteng
HC006	General Practitioner	Public	Western Cape
HC007	Psychiatrist	Public	Western Cape
HC008	Psychiatrist	Public	Gauteng
HC009	Psychologist	Public	Kwa-Zulu Natal
HC010	Psychologist	Public & Private	Gauteng
HC011	General Practitioner, Trainer	Private	Gauteng
HC012	Psychologist	Public	Gauteng

All interviewees were approached by email and provided with information about the background, methodology and objectives of the study, allowing the researchers to introduce the study and arrange an in-person or telephonic interview, depending on the interviewee’s availability and location. Thirteen professionals were contacted, and indicated interest, but only twelve proceeded to participate. Interviews took place at locations of the participant’s choosing, either at their place of work (for participants located in the wider Cape Town area) or by phone (for participants living in other parts of South Africa).

### Data collection

Two authors (AM and TM) conducted interviews. Both interviewers are employed as academic staff at their institution and have more than seven years qualitative research experience each. Both interviewers are female and cisgender. Rapport was easily established by the fact that the interviewers are researchers in a health science faculty and have intimate knowledge of the healthcare system and the field of specialisation of the providers interviewed. One of the interviewers (AM), a physician herself, had an existing professional relationship with most participants. The interviews were semi-structured, based upon a standardised interview guide, and conducted in a private conversation with the participant. Briefly, they ascertained the interviewee’s provision of services for transgender people; their use of ICD codes in service provision; the barriers and/or facilitators they experience in the provision of GAC; the guidelines that inform their provision of such care; and their understanding of the necessity of GAC. The interviews ranged between fifteen and sixty minutes in duration, were conducted in English, and were audio recorded for data accuracy. In two instances, follow-up interviews were conducted to clarify and/or expand upon an initial interview; otherwise, interviews were not repeated. Given that interviewees were practising healthcare providers with a busy schedule, we did not ask participants to read their transcripts for further comments.

### Data analysis

The interview recordings were transcribed and proofread. Additionally, the interviewing researchers (AM and TM) took notes during the interview. All data were analysed for thematic fields related to the research question by each researcher, making use of a content analysis framework. The principal researcher (AM) read all data in a comparative analysis to identify key themes around which further analysis was structured. Following this, the principal researcher and one co-investigator (SS) determined codes for all data and harmonized the key themes. Analysis was then done by one researcher (SS) using MSOffice (Word and Excel, Microsoft Corp.) and NVivo (QSR International) software for qualitative data analysis.

### Ethical and regulatory compliance

Approval for this research was obtained from the Human Research Ethics Committee of the Faculty of Health Sciences, University of Cape Town (HREC 857/2014). Participants were interviewed after giving written informed consent. In order to protect participants’ anonymity, we attribute information given by them through the interview codes listed in Table 1.

## Results

### Availability of gender-affirming care

The South African Department of Health has not issued any guidelines or policies on GAC, nor is transgender health recognized as a medical specialty. Six public hospitals in urban centres provide various components of GAC (Table 2); however, the waitlist, particularly for surgery, is long and growing in the public sector—up to 25 years at Groote Schuur Hospital in Cape Town, as, due to limited resources, the provincial Department of Health only allocates four theatre days a year for gender affirming surgeries (HC004) [26–28].

**Table 2:** Available gender affirming care in South Africa [26–28].

Facility	Location	Private/Public	Services Offered
Chris Hani Baragwaneth Hospital	Soweto, Gauteng	Public	Endocrinology
			Psychology
			Psychiatry
			Surgery
Helen Joseph Hospital	Johannesburg, Gauteng	Public	Surgery
Greys Hospital	Pietermaritzburg, KwaZulu Natal	Public	Endocrinology
			Psychology
Groote Schuur Hospital	Cape Town, Western Cape	Public	Endocrinology
			Psychiatry
			Surgery
Red Cross War Memorial Children’s Hospital	Cape Town, Western Cape	Public	Paediatric Endocrinology
			Child & Adolescent Psychiatry
Steve Biko Academic Hospital	Pretoria, Gauteng	Public	Endocrinology
			Psychiatry
			Surgery

Alternatively, psychosocial support, hormone therapy, and, to a much more limited extent, gender affirming surgeries are available through the private sector, although generally not covered by health insurance (HC004), as none of these procedures are recognised under the prescribed minimum benefits that regulate essential coverage [29]. Anecdotal evidence suggests that health insurances oppose claims for GAC on the grounds that they deem it ‘cosmetic’ [26, 28].

### The limited and discretionary nature of service provision

Interviewees reinforced that GAC is available in the public and private sector, but that access is limited to the urban centres of South African provinces with better infrastructure. The majority of interviewees worked in public hospitals. While a number of these public sector specialists also provide care in the private sector, those interviewees providing GAC exclusively in private care do so through their own private practices as GPs.

In KwaZulu-Natal, one psychologist and one endocrinologist at Grey’s Hospital in Pietermaritzburg are providing GAC. These two professionals are lobbying their hospital to recruit a surgeon to provide surgical services (HC009).

In Gauteng, GAC services are being provided at Steve Biko Academic Hospital in Pretoria, Helen Joseph Hospital in Johannesburg and Chris Hani Baragwaneth Hospital in Soweto. Professionals at the latter—all mental health professionals—formed a transgender health team over the last few years in response to a lack of guidance for trans patients or HCPs:

*It started off with one psychologist [who] was the first one to come and try and help with transgender patients*. *And because endocrine was being so difficult and access to hormones or even surgery or any other gender affirming kind of treatment […] we decided to start a little bit of a panel* (HC008).

The panel conducts preliminary assessments to determine the level of psychosocial support necessary for an individual, provides necessary mental health services, and facilitates access, often ad hoc, to the endocrinology and surgery departments (HC008, HC010, HC012).

In the Western Cape, GAC is available at the Red Cross War Memorial Children’s Hospital and Groote Schuur Hospital, both in Cape Town. At Red Cross, a private sector psychiatrist provides pro-bono services once a week and, through their advocacy, adolescents can also access paediatric endocrinology services or be referred for further care at Groote Schuur Hospital (HC001, HC002). Groote Schuur Hospital is the home of the Multidisciplinary Transgender Unit, which provides GAC through a team of specialists [26]. The Unit comprises psychologists and psychiatrists, endocrinologists, a surgeon, and a clinical social worker (HC003, HC004, HC007). Occasionally, it has also included a laser therapist and gynaecologist (HC007). One distinct feature of the Unit is its strong ties to local transgender community organizations, which help to inform its provision of services and clients’ access to the Unit (HC003, HC007). This successful model is owed to the initiative of a few dedicated HCPs:

*[The] clinic had been run since the 1980s but it was run in a very disjointed manner [by two doctors]*. *And then both retired and then there was a gap from about 2001 through to about 2009*. *[Patients] came through but they were seen by a variety [of professionals] and it just was a bit of a mess and we didn’t have a surgeon*. *[…] then 2009*, *we got together*, *told ourselves we have to do something about this*. *And then we got everyone […] we got a group*, *which hadn’t happened in other [places] so we were quite pleased […]*. *We got people from outside and social workers*, *[…] and it’s been working and we called that a transgender clinic and then the hospital accepted that as such*, *that we would do [provide GAC] and so that’s how we started* (HC007).

While access to GAC is largely concentrated in a few urban centres, there are also some GPs providing certain aspects of GAC on a case-by-case basis in a variety of healthcare settings across the country. Aware of the potential negative consequences of the lack of guidelines for access to GAC, one of the university-based professionals we interviewed had developed a pamphlet of basic guidance for such GPs (HC006). Such care is limited to psychosocial support and gender affirming hormone therapy, given that only tertiary institutions can conduct surgeries (HC009). In some cases, clients will travel to a tertiary institution for initial services and can then be monitored by a local GP or by the specialist from a distance (HC004, HC012). As a surgeon pointed out:

*The other thing that I would encourage the other guys to do*, *the psychiatrists*, *the endocrinologists*, *is make use of email*, *Skype*, *you know*? *They often don’t need to see the patient they just need to give insight to colleagues* (HC004).

Such long-distance care is largely discretionary and dependent on the will and knowledge of the HCP.

In addition to traditional healthcare services, the key informants all highlighted the role of community organizations—specific to either transgender or lesbian, gay, bisexual, transgender, intersex, and queer (LGBTIQ)—as integral to the provision of GAC. The work being done by Triangle Project and Gender DynamiX in Cape Town (Western Cape), Transgender Intersex Africa (TIA) and OUT in Pretoria (Gauteng), and Social, Health and Empowerment Feminist Collective of Transgender and Intersex Women of Africa (S.H.E.) in East London (Eastern Cape) has become integral to locating and accessing services (HC001-007, HC009-010, HC012). Additionally, these organisations are actively engaged in advocacy for the expansion of services for transgender clients by health professionals and institutions, and have contributed to improved services by directly providing training and information to HCPs about current best-practices in the provision of GAC (HC007, HC009). For example, Gender Dynamix has published three clinical guidelines on gender-affirming care adapted for primary care providers in the South African context [3, 30, 31]. These organisations, like the healthcare institutions providing GAC, are mostly located in urban centres (with the exception of S.H.E.) and whilst they may provide outreach to a wider area, their influence is largely evident in the urban healthcare facilities where they can constantly lobby and educate HCPs. For example, the Multidisciplinary Transgender Unit at Groote Schuur Hospital has had a long and successful partnership with Gender DynamiX, up to the inclusion of Gender DynamiX representatives at its review meetings (HC007), which has seen it take a truly multidisciplinary coordinated patient-centred approach [26].

### Clinical decision making

The interviewees referenced a variety of nationally and internationally produced guidelines and standards of care which guided their provision of GAC. All but two of the professionals explicitly acknowledged the WPATH Standards of Care as an important resource to their provision of care. Though the majority of these references were to the most recent SOC-7, some noted that they or colleagues they worked with are consulting prior editions (SOC-6).

In addition, service professionals also referenced guidelines put forth by the International Endocrine Society [32] (HC006, HC011); the Centre for Excellence for Transgender Health in San Francisco [33] (HC006); Callen-Lorde Community Health Centre in New York [34] (HC006, HC011); the Transgender Health Information Programme in British Columbia [35] (HC011); the Psychological Society of South Africa [5] (HC009); WHO [7] (HC001-005, HC007-009, HC011-012); APA [8] (HC001, HC003, HC004, HC007, HC009); Gender DynamiX [3, 30, 31] (HC006, HC009); and various academic journal articles (HC009, HC001).

With the exception of a position statement by the Psychological Society of South Africa [5] and guidelines by the community organization Gender DynamiX [3, 30, 31], there are no national guidelines for the provision of GAC and no state-recognized standards of care (HC001, HC002, HC004, HC012). Further, use of the international guidelines that the HCPs referenced as informing their practices are not mandated (HC001, HC002, HC008, HC009). Thus, HCPs are left to seek out guidance and best-practice guidelines in their own time and of their own volition.

A number of HCPs noted that the current system, whilst extremely uneven, can facilitate the pursuit of GAC for clients (HC002-004, HC007-009). Accessing services does cover the spectrum of services; however, access to mental health professionals, which form the basis of GAC in South Africa, is much more available than access to gender affirming surgeries. In part, this is a product of the knowledge and will of specific HCPs and resources available, including surgery theatre availability and plastic surgeons (HC004, HC007), but it is also reflective of the affordability and health insurance coverage of such procedures (HC004, HC005, HC007, HC008).

Some service professionals saw the lack of guidelines as somewhat advantageous in allowing for individualized and ‘creative’ care (HC001, HC003, HC004, HC010). This most commonly meant that service professionals could choose comparable non-transgender specific ICD or DSM codes that might better facilitate medical scheme payments for a service (HC007, HC010, HC011). It was also suggested by interviewees that the absence of guidelines allows for flexibility in their provision of care. For example:

*[…] I do treat them as guidelines every so often*. *I mean*, *I haven’t done*, *I haven’t said anyone must go for surgery early*. *But the gender affirming hormones and that*, *sometimes I think*, *you know*, *really*, *this kid’s ready*. *And I know [the plastic surgeon] has come and said to me ‘why must we wait until whenever’*. *Because it’s so distressing to the kid concerned* (HC001).

The public health sector in South Africa is extremely resource constrained [36], making pragmatic adaption of international guidelines important and necessary. This is also in line with the WPATH’s move towards context-specific guidelines in their most recent version:

*The nice thing I like about SOC-7*, *they finally recognise that the world is not American*, *so we do have national latitude so we’ve changed the guidelines in our clinic*. *You don’t need two independent psychiatrists with PhDs* (HC004).

Another reason these professionals gave for not concerning themselves with advocating for national guidelines was that to do so would detract from the GAC that they are already struggling to provide, often in unsupportive institutional contexts (HC008-010, HC012). Those HCPs who have committed themselves to serving transgender and gender diverse clients do so in a severely constrained environment, and, with their time at a premium, saw spending time on the development of guidelines as beyond their capacity and the scope of their jobs (HC001).

Some respondents were ambivalent about the role of national guidelines. In relation to the constraints imposed on reimbursement of care by medical aids, one psychologist noted, “[t]he care is the best you can give at the time” (HC007). At the same time, some service professionals wondered whether the absence of national guidelines was impeding access to funding by medical schemes (HC003, HC004). That is, having nationally derived guidelines that account for the contextually appropriate and available resources and services and which acknowledge the necessity of GAC could be useful in advocating for financial coverage (HC001, HC004).

The absence of national guidelines for HCPs has a number of additional implications. First, while the HCPs we interviewed had consulted a variety of documents, both within and across the specific sectors of GAC, these guidelines are not necessarily aligned. And, because of the constantly evolving standards of care for GAC, some HCPs knew of colleagues who used outdated guidelines, or used outdated guidelines themselves (HC004, HC007, HC009, HC010). As a result, different HCPs have different ideas of what constitutes appropriate GAC (HC001, HC004, HC010, HC012). Because GAC is an interdisciplinary process, this can create significant problems as clients move from one HCP to the next, as the following quote illustrates with an example about the revised requirements (from SOC-6 to SOC-7) in order to initiate hormone therapy:

*We [the psychiatrists] have got a lot of [patients] coming back*, *with endocrines [the endocrinologist] saying the person is not coming dressed [aligned with their identified gender]*, *and we say*, *“but SOC-7 doesn’t say that so…’' But it is an issue because at one stage it was that SOC-6 did have it [as a requirement]* (HC007).

Moreover, interviewees noted an unwillingness and/or inability of HCPs in the provision of appropriate GAC as another hindrance to access for clients (HC005, HC007, HC008, HC010, HC011). That is, psychologists and psychiatrists that make a diagnosis of GID/GD and who refer their client to an endocrinologist must first find an endocrinologist who is willing and able to provide gender affirming hormone therapy. A number of interviewees (HC001, HC005, HC009, HC010, HC012) felt that the absence of guidelines meant that they spent a significant amount of time advocating for access to GAC for their clients, both with other health professionals and health institutions:

*[…] the transgender services aren’t necessarily completely formalised*. *They’re still in the process of developing […] in the public healthcare system*. *I think across the country*. *So*, *in that sense*, *I think you need to be quite sensitive to how you actually deal with stuff and how you deal with the professional relationships* (HC012).

Lastly, as discussed above, the absence of national guidelines or protocol for the provision of GAC often required that service professionals be ‘creative’ in order to get their client the service they needed (HC003, HC007, HC010, HC011). As one interviewee pointed out, although this could facilitate access to GAC for some lucky clients, for other clients it implies a barrier to care which needs to be addressed:

*The part that bothers me around coding creatively is*, *are you coding creatively because you have to get around obstacles*? *So that your client can get the care they need*? *And I have a problem with that; because often the obstacles that you are trying to get around are obstacles that shouldn’t be there*. *Because they are obstacles informed by not up-to-date reasons and knowledge around the issue*, *or it’s informed by prejudice*. *So my natural activist is*, *no I don’t want to have to go around things to get for my client I want you to remove what is in the way […] of my client being able to get the care that they deserve* (HC003).

### Professional networks

A natural consequence of the lack of structure and guidelines for providing GAC in South Africa is the limited number of informed and willing GAC professionals (HC009, HC011). As has previously been discussed, the vast majority of known GAC professionals and all of those interviewed for this study are located in urban centres of the Gauteng, KwaZulu-Natal, and Western Cape provinces. Thus, clients seeking GAC who reside in rural settings, or in one of the other six provinces, will often be required to travel to access care (HC002, HC009, HC012). But this has implications for the quality of services they receive over the long term. One service professional noted:

*[…] we’ve had patients from mostly Gauteng but also more rural areas*, *even though that makes it quite hard actually to follow up on services and things like that*. *Gauteng or [Johannesburg] is a bit better*. *But yeah*, *we do see people from the more rural governances and so on*, *even though it’s not ideal […]*. *Because*, *for instance*, *it’s hard to find professionals in the rural areas that are sensitized to transgender issues or who are sensitized to gender dysphoria* (HC012).

Even within those urban centres, though, it can be difficult to locate a HCP who specializes in or is even comfortable providing GAC. Many of the interviewees noted the importance of transgender advocacy and LGBTIQ community organizations in providing information and referrals to GAC service professionals (HC003, HC005, HC007, HC010, HC012). Based upon our interviewee’s responses, it would appear that clients’ ability to access GAC can rely heavily on their initial point of contact. That is, if someone is fortunate enough to have access to the LGBTIQ community or a GP with knowledge of transgender health, they are likely able to refer them to a doctor who provides GAC. And once that individual has seen that doctor, they often are part of a community of professionals—or at least know of other service professionals—providing complimentary components of GAC (HC001, HC005, HC007). As the following quote illustrates, though, it is rare to find a GP who has adequate knowledge, and if GPs are knowledgeable about referral pathways for GAC, they know so because of their initiative to seek out more information:

*When we get [referrals] from GPs*, *it’s usually they’ve done the research or spoken to someone and said ‘who do we go to’*, *so they sort of know who they need to refer to* (HC007).

What was clear from the interviewees was the importance of professional networks—the connection and sharing of information between GAC service professionals (HC001, HC004, HC007). The informal nature of GAC in South Africa both requires and fosters a collaborative approach to educating, providing, and advocating for GAC (HC009, HC012). This is seen in the formation of transgender units within one of the tertiary hospitals in South Africa, as well as in the tight-knit relations between professionals in the GAC community (HC001, HC003, HC004, HC007, HC008, HC010, HC012). A Cape Town-based HCP felt that, in addition to referrals from GPs, ten percent of their referrals came from Gender DynamiX, ten to twenty percent from other physicians at Groote Schuur Hospital, and sixty percent from a single clinical social worker with the Triangle Project (HC007).

Gender DynamiX also hosts a biennial conference for the transgender community, which has been a significant venue for connecting GAC service professionals across the country and educating them on best practices and updated international guidelines. As one interviewee noted:

*[…] we understand that there are people like that doing work and when Gender DynamiX held a couple of conferences*, *a lot of people pitched up*. *So it was quite*, *it was nice that people in the business*, *we thought we were quite alone or we were feeling alone and we suddenly find other people also saying that there are a lot of people there and it’s hard to connect them* (HC007).

### Challenges to the provision of gender affirming care in South Africa

There are a number of challenges that HCPs face in their efforts to deliver GAC in South Africa. The first of these starts with the training they receive in their respective specializations, as transgender health-related topics are largely absent from health sciences curricula (HC005, HC009, HC011, HC012) [37]. Thus, for the HCPs interviewed in this study, the education they have acquired regarding the provision of GAC is the result of individual interest and initiative. However, not all healthcare professionals will have the ability to take this on:

*So as to find someone willing or interested to learn*. *Because what I found is people are so overwhelmed with their work*. *There is not a lot of space*. *So […] I can see the patient load*. *To bring in a very minority group that needs healthcare services has created quite a challenge* (HC009).

For many HCPs, their gateway to GAC was a personal connection to the transgender and gender diverse community (HC009, HC011). For others, it was a specific patient or another HCP that initiated their pursuit of information (HC003, HC005, HC010). Either way, seeking information on GAC occurs out of HCPs’ own volition, if and when that volition is present. For example, most HCPs said they use the WPATH, or other preferred guidelines, because of referral by other HCPs, NGOs, or because of their own research initiative.

The rarity of that engagement with transgender health-related topics and GAC has implications for both individuals seeking and professionals trying to provide GAC in South Africa. Because of the interdisciplinary nature of GAC, HCPs and clients rely on a number of health professionals, from GPs to psychologists, endocrinologists, pharmacists, and surgeons. Thus, one professional’s ability to provide a service often depends upon another professional’s ability and willingness to fulfil that service (HC005, HC007, HC008, HC010), which is where the previously discussed professional networks become so integral. To this end, a number of HCPs spoke of recruiting and advocating for GAC within institutions (HC004, HC007, HC008-010). And, when they engaged people and institutions for the first time on an issue with which they lack familiarity and comprehension, it could be a prolonged effort:

*[…] we are also trying to convince Greys [Hospital] to do [surgery]; that is our most important thing that we’re trying to push for and we’re still in process*, *a long educational route*. *Last year was Gender DynamiX—we did a kind of awareness program at Greys*, *as well*, *where we had a doctor come in and where*, *well*, *I presented from this side*, *then we had to*, *I think it was last*, *no*, *it was the previous year*, *2014*, *we had that as well*. *So it’s basically education* (HC009).

Beyond the availability and accessibility of GAC professionals, professionals’ ability to provide services, and clients’ ability to fulfil these are often impeded by South Africa’s funding mechanisms (HC002-005). In the public sector, this is impacted by resource constraints. As one interviewee noted, the public sector hospital they work at is allocated a budget—based purely upon the number of patients seen—which is only ever adjusted for inflation (HC007). And because that funding is from the provincial health department, only residents of that province can access those services (HC001). The limited funding then has implications on how much of a service can be provided. For example, the surgeon in the Multidisciplinary Transgender Unit at Groote Schuur Hospital can only perform two to three gender affirming genital surgeries per annum based upon the theatre time the hospital will allocate towards such procedures (HC004, HC007) [26, 28].

Privately, access to funding for GAC services is equally challenging. Access to coverage of services is highly dependent upon the type of service, with psychology and psychiatry services being more commonly covered than gender affirming hormone therapy and gender affirming surgeries (HC004, HC007). Inherent to this challenge is the role that coding plays in billing for medical services, where the use of ICD’s F64 codes for diagnoses of GID–which may be specified under diagnoses for Transsexualism (F64.0), Duel role transvestism (F64.1), GID of childhood (F64.2), Other GIDs (F64.8), and GID, unspecified (F64.9) [7]–or DSM’s codes for diagnoses of GD–which may be specified under diagnoses for Gender Dysphoria in Children (302.6) or Gender Dysphoria in Adolescents and Adults (302.85) [8]—can result in non-coverage. Resultantly, health professionals resort to ‘creative coding’ to help their clients’ achieve coverage for the required services (HC003, HC007, HC010). In this context of medical aids refusing coverage, HCPs also suggested their provision of pro bono services (HC002, HC005, HC010), but this is not a viable approach for all GAC in South Africa. In addition, it does not address the problem of discrimination experienced by transgender South Africans in accessing health services, another issue raised by HCPs and noted in the literature (HC002, HC003) [23–25]].

Such discrimination poses a number of additional challenges to the provision of GAC. It is often difficult to find a willing HCP to provide GAC and sometimes even HCPs who are open to providing GAC do not have the necessary knowledge (HC005, HC010). This highlights the need for training on transgender health and GAC. And, as one HCP asserts, this is tied to the absence of national guidelines:

*[…] you know if you see a consultant or you see someone that is against transitioning or hormone replacement therapy*, *then [the client will] be blocked*. *So*, *that’s our biggest problem and barrier—trying to get a uniform system with uniform rules that everybody in our country would follow*, *which would make it so much easier* (HC008).

Moreover, HCPs felt that a state-sanctioned resource for patients and HCPs, which recognized GAC as appropriate and necessary might help combat some of the prejudice that transgender and gender diverse people face in the health system and beyond (HC002-004).

## Discussion

The current provision of GAC in South Africa is inconsistent at best and non-existent at worst. While, technically, both the public and private health systems provide these services, there are disadvantages to both. As Klein recognizes, given that “income is still intimately connected with gender and skin colour, the situation is especially difficult for persons who did not grow up categorized as white males—but private care is much too expensive even for many who have been categorized this way” [20]. Thus, while GAC is theoretically available to South Africans, practical access is unequal, can be extremely protracted, and is limited for all but the wealthiest.

Though the South African health professionals that we spoke to have been resourceful in seeking out guidance in their provision of GAC, the absence of formal guidelines has a number of consequences for both clients and HCPs. Significantly, whether GAC is provided at all, and how, is often discretionary, and dependent on individual interest, effort and networks. As willing professionals may refer to any of various international and sector-specific guides, when it is provided, care is highly varied. This is compounded by the interdisciplinary and sometimes highly specialized nature of GAC, where one client—depending on the extent of services they require to help fulfil their gender affirmation—may rely on the services of a mental health professional, endocrinologist, pharmacist, and surgeon. Resultantly, without a central process to inform all HCPs, clients’ access to care can be exceedingly heterogeneous.

Yet, the consequences of withholding GAC from transgender individuals who wish to access it are severe: a study in Ontario, Canada found that the status of individuals’ medical gender affirmation had a significant positive correlation with suicidality, where participants who were planning a medical gender affirmation had either seriously considered (55%) or had attempted (27%) suicide [16]. This is in contrast to those who had seriously considered (23%) and who had attempted (4%) suicide amongst participants who had either completed their gender affirmation or were not planning to medically affirm their gender at all [16]. Likewise, informed by evidence put forth by WPATH in its SOC-7 [6] and an expansive body of research (such as [38–41]), the American Psychiatry Association has recognized that the “lack of access to care adversely impacts on the mental health of transgender and gender variant people, and both hormonal and surgical treatments have been shown to be efficacious in these individuals” [15]. In light of this evidence, and in addition to its constitutional provisions on the right to access to healthcare, South Africa’s health system also has an ethical obligation to increase access to GAC.

Within the community of GAC-providing health professionals, the knowledge of what colleagues were willing to provide and believed to be appropriate gender affirming care begins to act as a guideline. That is, when one HCP knows how another HCP approaches GAC, they begin to shape their service provision accordingly. For example, while some endocrinology guidelines suggest six months of gender affirming hormone therapy prior to certain surgeries, one endocrinologist noted their awareness of other professionals’ flexibility in this regard. Thus, in the absence of national guidelines, the various actors that come together to provide GAC create a self-regulating system of sorts. While this can have positive outcomes for some clients where health professionals seek to facilitate access to GAC, this can equally mean that others are constrained in their efforts to access care. For example, evidence from healthcare service provision to other stigmatized groups in South Africa, including sexual minorities and teenagers seeking sexual and reproductive health services, show that HCP discretion significantly impedes access to services [42].

Further, where access to services becomes dependent on certain HCPs, it cannot be considered sustainable—those few HCPs who are currently providing GAC won’t always be available, as they retire, experience occupational fatigue, or incur other obstacles to their continued practice. Currently, given the absence of GAC from health education curricula [37, 43], there is nothing in place to ensure their replacement. Moreover, there is little long-term viability to accessing the necessary services when these are only affordable due the pro bono GAC that some HCPs elect to provide. And, because trans people are even more stigmatised and information is comparatively less readily available [44], this means that personal discretion plays an even more central role in in the provision of GAC, with the effect that transgender individuals experience routine discrimination and exclusion in South Africa’s public health facilities [23, 24].

All of which is predicated, however, on the assumption that HCPs are comfortable engaging in GAC. While we have been able to speak with a variety of knowledgeable and engaged providers of GAC, these individuals are in the minority in their respective professions. Many of the interviewees discussed a variety of scenarios where access to care for transgender clients was impeded by individuals in the health sector who had no or limited awareness or training regarding gender affirming care. This is not surprising, given that transgender health concerns in general, and GAC in particular, are rarely, if ever, covered in health professions education in South Africa [37, 43]. However, HCP education is a crucial step towards increasing access to GAC, as education around sexual and gender minority health increases HCPs’ willingness to provide services [23, 45], and also equips health professionals with the tools to become advocates for their patients. Once again, national policy guidelines are key, as without them it is unlikely that many HCPs will access education on trans health and GAC. These should take into account the suggested reclassification of ‘gender incongruence’ in the ICD-11 [11], as well as contemporary debates around depathologisation and access to care [10]

We believe that our findings are not unique to South Africa. Research, even if scarce, shows that the obstacles for GAC that our findings highlight are global: health professions education does not adequately cover GAC [37, 43, 46]; national health policies often do not acknowledge the existence of transgender individuals beyond their needs for HIV prevention and treatment [47]; and with the exception of some Canadian provinces and some insurances in the US, health insurance schemes do not cover GAC-related procedures [48]. Studying the ways in which HCPs provide GAC in the absence of formal policies, guidelines, or health professions education is therefore relevant to all contexts where GAC is marginalised in health policy and planning.

## Conclusion

We believe that nationally approved guidelines, acknowledging the necessity of GAC, are an important step toward broadening and improving access to GAC in South Africa. It would provide a state-sanctioned mandate for the provision of GAC and clear clinical guidelines to standardize care, homogenizing and expanding the provision of GAC in the public sector beyond existing urban hubs. Additionally, it would provide an impetus for financial coverage for concomitant GAC within the private sector, improving access for those able to procure private medical insurance.

## Supporting information

S1 File Interview guide. Interview guide for healthcare provider interviews.
(PDF)
