# Supplementary material for: “The care is the best you can give at the time”: Health care professionals’ experiences in providing gender affirming care in South Africa
Source: PLoS One. 2017 Jul 12;12(7):e0181132. doi: 10.1371/journal.pone.0181132 (PMC5507544; doi:10.1371/journal.pone.0181132)
Supplement: S1 File — Interview guide for healthcare provider interviews. (PDF) [file pone.0181132.s001.pdf]

Gender-affirming care and transgender health-specific ICD coding practices: key informant interviews

Thank you for agreeing to talk to me about gender-affirming care and ICD coding practices in transgender health care. My name is _____________, I work at the Gender, Health and Justice Research Unit at the University of Cape Town, and I am one of the main researchers of this project.

Key information

- Health care provider: *Specialisation and name (if agrees to be named)*

Questions

1. What services do you provide for transgender people? Where do you provide these services? (geographical area, public/ private sector, level of care) How did you start providing these services?
2. Based on your professional experience, what are the barriers to and facilitators of providing transgender health care in South Africa?
3. In the absence of nationally approved treatment guidelines for transgender health, what guidelines do you use?
4. Can you distinguish between transgender medical procedures that might be classified as clinically appropriate but not medically necessary?
5. Do you make use of ICD codes for the transgender-specific services you provide?
    a. If yes, which ICD codes do you use for transgender-related procedures, treatment and medication?
    b. If yes, what have been your experiences with using ICD codes for transgender-related procedures, treatment and medication? With patients? With medical aid schemes? With colleagues?
    c. If no, why not?
6. When selecting an ICD code, how do you decide whether a procedure is cosmetic or reconstructive?
7. If WHO changed the ICD codes related to transgender health care, what would you recommend based on your professional experience?
8. The Prescribed Minimum Benefits appear to exclude medical procedures related to transgender health; can this be linked to the ICD coding? Is there a medical argument to be made to include gender-affirming procedures in the PMB?
9. Have you provided expert support/ testimony for transgender patients accessing other public services (ie. applying for ID documents)? Did you use ICD codes for this?
    a. If yes, why, and what was the outcome?
    b. If no, why not, and what was the outcome?
